# The protective role of montelukast against intestinal ischemia-reperfusion injury in rats

**DOI:** 10.1038/srep15787

**Published:** 2015-10-26

**Authors:** Shenbao Wu, Xuxing Zhu, Zhonghai Jin, Xiuping Tong, Liqin Zhu, Xiaofei Hong, Xianfei Zhu, Pengfei Liu, Weidong Shen

**Affiliations:** 1 Department of Gastroenterology, the Affiliated Yiwu Hospital of Wenzhou Medical College, Yiwu 322000, PR China; 2 Department of Gastroenterology, the Affiliated Jiangyin Hospital of Nantong University Medical School, Jiangyin 214400, PR China

## Abstract

Several drugs are effective in attenuating intestinal ischemia-reperfusion injury (IRI); however little is known about the effect of montelukast. Fifty rats were randomly assigned to 3 groups: model group (operation with clamping), sham group (operation without clamping), and study group (operation with clamping and 0.2, 2 and 20 mg/kg montelukast pretreatment). Intestinal ischemia-reperfusion was performed by occlusion (clamping) of the arteria mesenterica anterior for 45 min, followed by 24 h reperfusion. Intestinal IRI in the model group led to severe damage of the intestinal mucosa, liver and kidney. The Chiu scores of the intestines from the study group (2 and 20 mg/kg) were lower than that of the model group. Intestinal IRI induced a marked increase in CysLTR1, Caspase-8 and -9 expression in intestine, liver and kidney, which were markedly reduced by preconditioning with 2 mg/kg montelukast. Preconditioning with 2 g/kg montelukast significantly attenuated hepatic tissue injury and kidney damage, and decreased plasma interleukin-6 (IL-6) and tumor necrosis factor-α (TNF-α) levels in plasma after intestinal IRI. In conclusion, preconditioning with montelukast could attenuate intestinal IRI and the subsequent systemic inflammatory response in rats.

Intestinal ischemia is generally the result of arterial occlusion by thrombi or emboli and, more frequently, by nonocclusive processes, such as in situations of low mesenteric flow^1^,^2^. The arteries most compromised by obstruction are the celiac artery, superior mesenteric artery and inferior mesenteric artery^3^.

Ischemia leads to hypoxia, which initiates a series of events primarily related to the activation of platelets and the release of vasoconstrictive mediators, which further restricts blood flow to the ischemic area. The degree of tissue injury is further enhanced and accelerated by reperfusion, which is not only associated with local changes, but also with systemic changes^4^,^5^,^6^. Moreover, it has been demonstrated that the 5-lipoxygenase (5-LO) pathway plays a significant role in the pathophysiology of intestinal IRI^7^,^8^. Cysteinyl leukotrienes (CysLTs) are produced from arachidonic acid through the 5-LO pathway and act on the CysLT1 and CysLT2 receptors^9^,^10^,^11^.

Montelukast is used for the maintenance treatment of asthma and to relieve symptoms of seasonal allergies as a selective reversible CysLT1 receptor antagonist. CysLT1 receptor antagonists or biosynthesis inhibitors ameliorate ethanol-induced gastric mucosal damage^12^ and impaired wound healing. Recent studies have shown that montelukast has an antioxidant effect in testicular injury and also reduces renal damage^13^. Furthermore, the beneficial effects of montelukast have also been reported in various experimental models of inflammation^14^,^15^,^16^. To our knowledge, few studies have investigated the protective/therapeutic effects of montelukast in intestinal IRI and the mechanisms involved. Therefore, the current study was designed to explore the protective effects and possible mechanism of montelukast against intestinal IRI in rats.

## Results

### Rats preconditioned with montelukast exhibit significantly reduced damage to the intestinal mucosa

**Figure 1:**
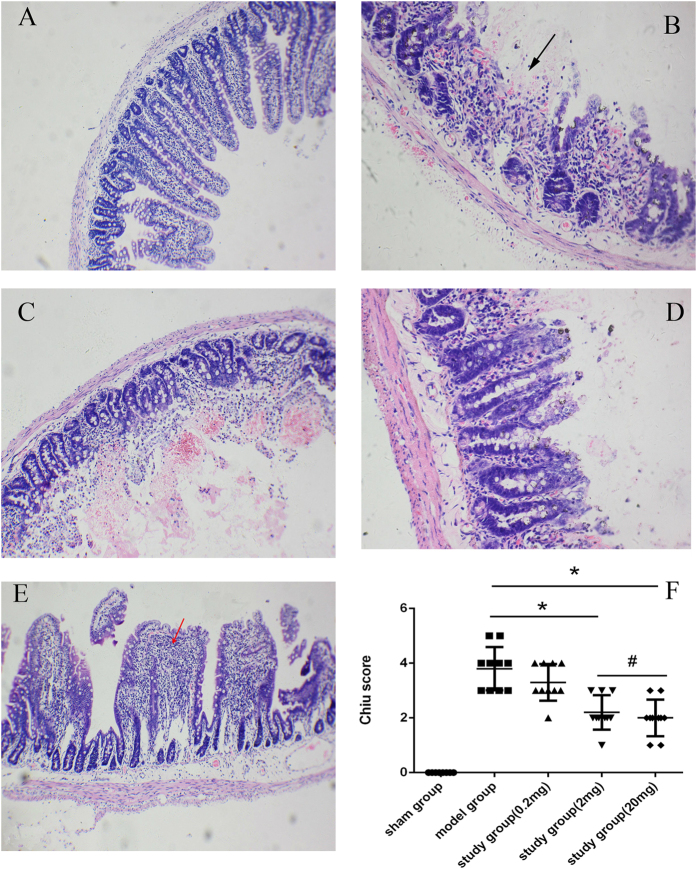
Montelukast protects against intestinal injury after intestinal ischemia-reperfusion injury (IRI). Representative photomicrographs (100×) of sham-operated group (**A**), model group (**B**), and study group with 0.2 mg/kg (**C**), 2 mg/kg (**D**), 20 mg/kg (**E**) montelukast. Black arrow denotes areas of bleeding and necrosis, red arrow denotes areas of neutrophil infiltration. Intestinal histology was evaluated using the Chiu score ((**F**); scale 0–5). *P < 0.05.

In Fig. 1, we demonstrate the protective effects of montelukast in the small intestine. Intestinal IRI in the model group led to severe damage to the intestinal mucosa (Fig. 1B). Compared with the model group, rats preconditioned with montelukast (2 and 20 mg/kg) prior to intestinal IRI had markedly reduced intestinal ischemia reperfusion injury (Fig. 1D,E) and had a significant reduction in Chiu score (Fig. 1F, *P* = 0.017 and 0.009). However, there was no obvious difference in Chiu score between the 2 and 20 mg/kg groups (*P* = 0.106).

### CysLTR1 protein and mRNA expression is attenuated following pretreatment with the 2 mg/kg dose of Montelukast

**Figure 2:**
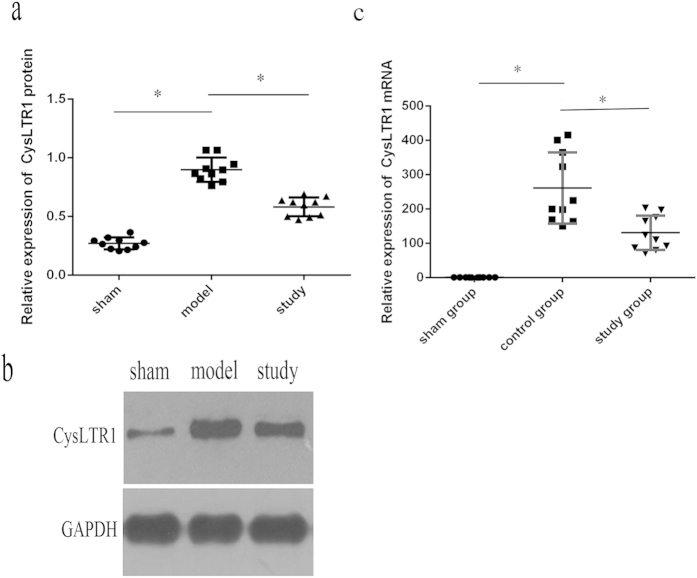
Cysteinyl leukotrienes receptor-1(CysLTR1) protein and mRNA expression after intestinal IRI. CysLTR1 protein expression was detected by western blotting (**a**,**b**) and mRNA expression by real-time PCR (**c**). *P < 0.05.

As expected, the expression of the CysLT1 receptor in the intestinal tissue of sham-operated rats was low. Intestinal IRI induced a marked increase in CysLTR1 protein expression in intestinal tissue (*P* = 0.006), which was dramatically reduced following preconditioning with montelukast (*P* = 0.014). Similar results were obtained for CysLTR1 receptor mRNA expression (Fig. 2).

### Preconditioning with montelukast (2 mg/kg) reduces intestinal apoptosis after IRI

**Figure 3:**
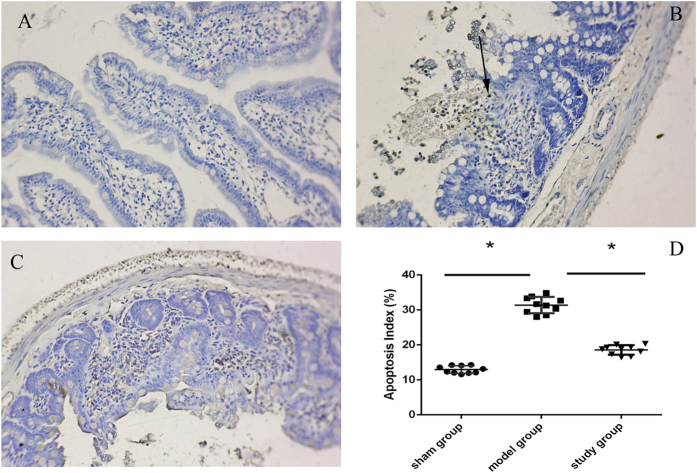
Montelukast protects against apoptosis after intestinal IRI. Representative photomicrographs illustrating apoptotic nuclei (TUNEL fluorescence staining) in the intestine of the sham group (**A**), model group (**B**), and study group ((**C**), montelukast, 2 mg/kg) (representative of four experiments, 100× magnifications). (**D**) Histogram of the AI in the ileum of rats. *P < 0.05. Rats after intestinal IRI showed many TUNEL-positive cells in the distal tips of villi in the small intestine (Fig. 3B, arrow denotes TUNEL-positive cell).

**Figure 4:**
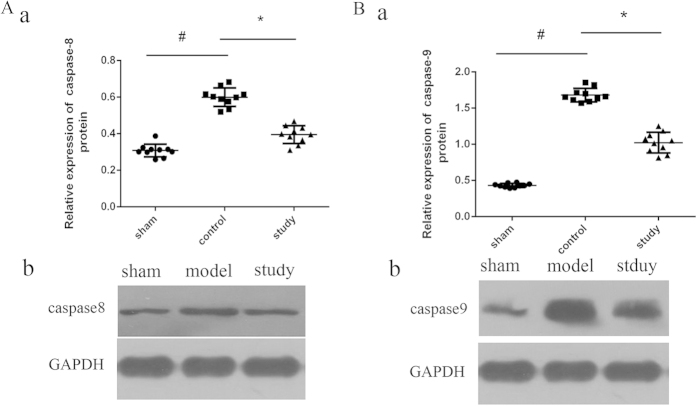
Caspase-8 and caspase-9 protein expression after intestinal IRI. Protein expression was detected by western blotting. *P < 0.05.

Apoptosis in intestinal tissue was detected using TRITC staining and observed under an ImageScope (Aperio, Vista, USA). Five random fields of view (400× magnification) were taken and positive cells were counted. The apoptosis index (AI) = the average number of apoptotic cells/200 × 100%. The assessor was blinded to the treatment groups. As seen in Fig. 3, the AI decreased from 33.33 ± 6.76% in the model group to 17.11 ± 5.58% with 2 mg/kg montelukast preconditioning (*P* = 0.019). Thus, preconditioning with montelukast reduces apoptosis in intestinal IRI. As evident in Fig. 4, the expression of caspase-8 and caspase-9 markedly decreased with montelukast preconditioning (*P* = 0.021 and 0.018).

### Montelukast (2 mg/kg) protects against acute hepatic and kidney injury after intestinal IRI in rats

**Figure 5:**
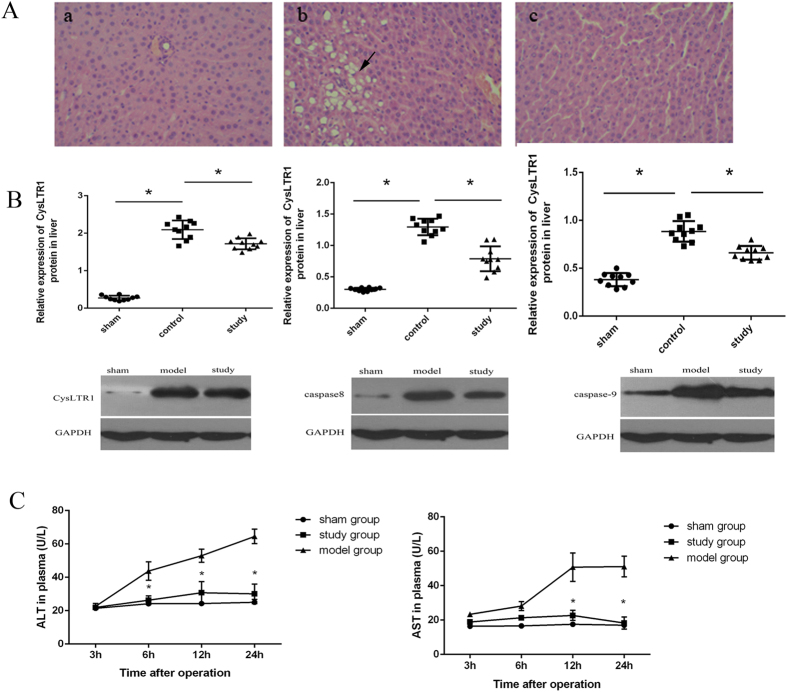
Montelukast reduces liver injury after intestinal IRI. Representative photomicrographs of livers from rats in different treatment groups ((**A**); H&E staining, 200× magnification. **A**-a, sham group; **A**-b, control group; **A**-c, study group). Arrows indicate vacuolisation. CysLTR1, caspase-8 and caspase-9 protein expression in liver from different groups. Plasma ALT and AST levels were measured in rats at various times after intestinal IRI (**C**).

**Figure 6:**
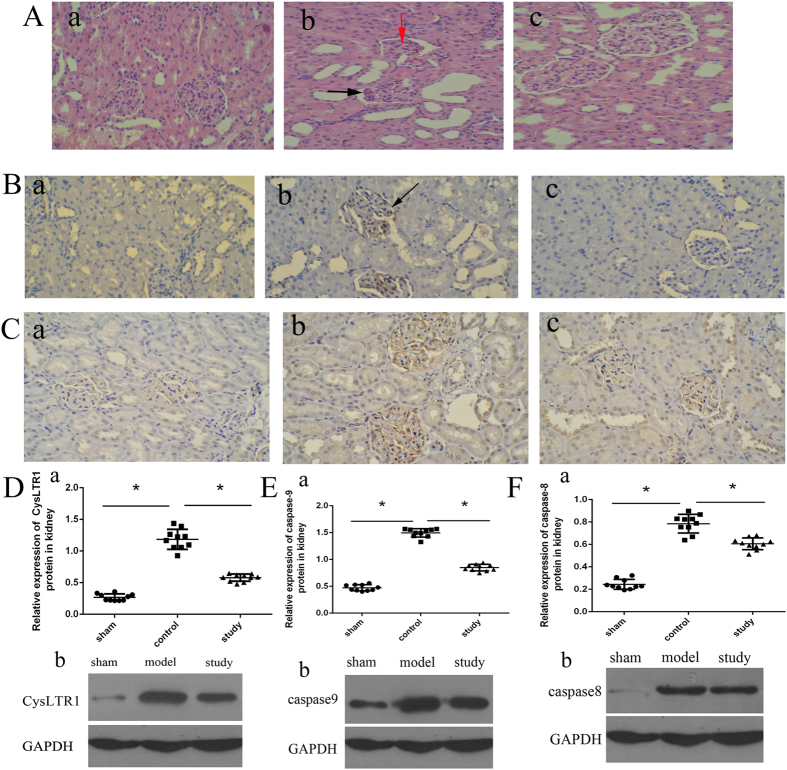
Montelukast reduces kidney injury after intestinal IRI. Representative photomicrographs of kidneys from rats in different groups (**A**-a, sham group; **A**-b, control group; **A**-c, study group. H&E, 200× magnification). highlighting tubular simplification, hypereosinophilia and glomerular atrophy (A-b, down and red arrows). Expression of NGAL in renal tissues in different treatment groups ((**B**), **B**-a, sham group; **B**-b, control group; **B**-c, study group). CysLTR1 protein expression in the kidney from different treatment groups by immunohistochemistry and western blotting ((**C**,**D**). **C**-a, sham group; **C**-b, control group; **C**-c, study group). Protein expression of caspase-8 and caspase-9 in the kidney (**E**,**F**). *P < 0.05.

Twenty-four hours after intestinal IRI, rats developed significant hepatic and renal dysfunction as indicated by a rise in plasma ALT (*P* = 0.000) and AST levels (*P* = 0.000) (Fig. 5C), and the expression of NGAL in renal tissue (Fig. 6B-b) was above sham values. In the liver, intestinal IRI resulted in marked vacuolisation and leukocyte infiltration (Fig. 5A-b, left arrows denote vacuolisation area) compared with sham-operated rats (Fig. 5A-a). With montelukast preconditioning, there was an obvious decrease in vacuolisation and leukocyte infiltration after intestinal IRI (Fig. 5A-c). The expression of CysLT1, caspase-8 and caspase-9 also decreased with montelukast preconditioning (*P* = 0.034, 0.015 and 0.039 respectively). In the kidney, there was increased proximal tubule simplification (Fig. 6A-b, right black arrows), proximal tubular hypereosinophilia/single cell necrosis, and glomerular atrophy (Fig. 6A-b, down red arrows). The expression of CysLT1 decreased with montelukast preconditioning (Fig. 6C,D), whereas caspase-8 and -9 expression were similar (Fig. 6E,F).

### The induction of IL-6 and TNF-α are attenuated by pretreatment with with the 2 mg/kg dose of Montelukast

**Figure 7:**
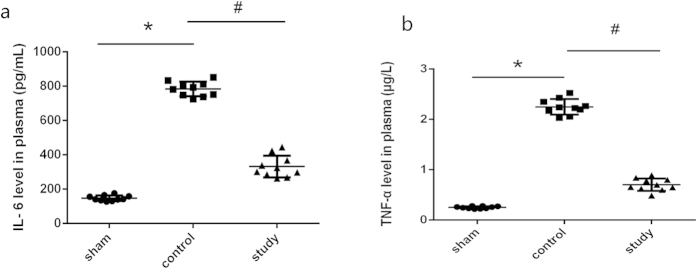
Plasma interleukin-6 (IL-6) and tumor necrosis factor-α (TNF-α) levels after intestinal IRI in rats. Blood was obtained immediately after serum creatinine reperfusion, and IL-6 and TNF-α levels were assessed by ELISA. *P < 0.05.

As expected, the levels of IL-6 and TNF-α in plasma from sham-operated rats was low and intestinal IRI induced a marked increase in plasma IL-6 (to 782 pg/mL, approximately 5-fold) and TNF-α (to 2.248 μg/L, approximately 9-fold), which were markedly reduced (282 pg/mL and 0.701 μg/L, respectively, *P* = 0.000) by preconditioning with montelukast (Fig. 7).

## Discussion

Intestinal IRI is a troublesome clinical disease with a high mortality rate, despite surgical intervention^17^. Ischemia and reperfusion injury has different pathophysiological features with the major mucosal injury happening during the reperfusion phase^18^,^19^. Thus, using pharmacological agents is an important strategy for preventing intestinal ischemic injury during intestinal IRI^19^,^20^,^21^,^22^,^23^.

Montelukast has been shown to have a neuroprotective and antiapoptotic effect in an IRI mouse model by acting as a CysLT1 receptor antagonist at concentrations of 0.1**–**1.0 mg/kg^24^,^25^,^26^, and these effects are related to the inhibition of neutrophil accumulation, lipid peroxidation and pro-inflammatory cytokine release. Genovese *et al.*^27^ also showed that montelukast can reduce spinal cord inflammation, neutrophil infiltration and tissue injury. Recently, Erdem *et al.* from Turkey reported on the protective effects of montelukast and hypericum perforatum against intestinal IRI in rats^28^, and this effect may be due to its anti-apoptotic properties and the increased expression of malondialdehyde, myeloperoxidase, glutathione, and cardiotrophin-1^29^. However, the detailed signalling mechanism involved in the preventive effects of montelukast against intestinal IRI remains unclear. In addition, how montelukast prevents subsequent systemic inflammatory responses and distant organ damage, such as to the liver and kidney, during intestinal IRI in rats has not previously been established. Our study aimed to explore the role of montelukast on reducing intestinal IRI, including injury to the liver and kidney, in rats, and the possible signalling pathway involved.

We used three doses of montelukast (0.2, 2 and 20 mg/kg) to explore the effect on intestinal IRI. Intestinal morphological changes were observed by microscopy. Submucosa villi shrinkage, haemorrhagic necrosis, obvious inflammatory cell infiltration, muscle layer thickness changes, and bleeding were observed in rats from the model group, demonstrating that intestinal IRI could lead to the destruction of intestinal mucosa structure. The results demonstrated that 2 mg/kg montelukast could markedly alleviate the degree of damage in intestinal lesions. Further protection was not observed by increasing the dose. Thus, we explored the mechanism of action of 2 mg/kg montelukast against intestinal IRI. Montelukast is used for the maintenance treatment of asthma at a recommended dose of one 10-mg tablet/day for adults (approximately 0.2 mg/kg). In our study, the effective dose was 2 mg/kg, which is 10 times greater than that used regularly in humans. However, our basic study was conducted in rats, and a further suitable dose for preventing intestinal IRI, and the possible side effects associated with such a high dose requires further research.

Several studies have shown that the protective role of montelukast in IRI is related to the inhibition of apoptosis^29^,^30^,^31^. Our data also revealed that IRI can significantly increase intestinal cell apoptosis, suggesting that CysLTs/CysLTsR1 can promote intestinal apoptosis, and that apoptosis is involved in the mechanism of intestinal IRI^31^,^32^. With the application of montelukast, apoptosis was reduced in tissue and the expression of caspases-8 and -9 decreased, indicating that montelukast inhibits caspase-8 and -9 mediated pathways of apoptosis in intestinal IRI.

Intestinal IRI not only leads to injury in the intestine, but affects distant organs such as the liver and kidney^6^. In our study, montelukast reduced injury in both the liver and kidneys after intestinal IRI, as evidenced by improved histological findings and markers of organ function (plasma ALT, AST and NGAL levels). Preventive strategies are most effective when started before oliguria and elevated serum creatinine are evident, both of which are delayed and unreliable markers of kidney damage. A new promising, early biomarker is NGAL, which is believed to participate in the regeneration process. NGAL is an important biomarker of acute kidney injury (AKI) and acute tubular necrosis and correlates well with the degree of renal dysfunction^6^.

Inflammation is another important contributor to the exacerbation of IRI^18^, and we detected IL-6 and TNF-α as markers of the inflammatory response^20^. The role of montelukast to reduce IL-6 and TNF-α has not been previously reported in intestinal IRI. We found that montelukast markedly reduced systemic IL-6 and TNF-α after intestinal IRI, indicating that montelukast may protect not only the organ affected by the ischemic insult, but also blunt the systemic inflammatory response. Thus, montelukast may be of potential benefit in attenuating the systemic consequences of intestinal injury^32^.

Based on this evidence, we postulate that montelukast can directly protect the liver and kidneys after intestinal IRI by reducing the production and release of IL-6 in the small intestine, thereby reducing the systemic inflammatory effects in distant organs such as the liver and kidney. In addition, we found that montelukast could inhibit the expression of CysLTR1 in the liver and kidney. However, it is difficult to determine in an *in vivo* study whether the protective effect is mediated via direct inhibition of CysLTR1 signalling or via systemic effects such as modulation of IL-6 and TNF-α release. Based on the available evidence, it is likely that montelukast has multiple effects, including both direct cytoprotective effects in the intestine with inhibition of caspase pathways, as well as systemic anti-inflammatory mechanisms.

In conclusion, we demonstrated that montelukast could reduce intestinal mucosal injury and apoptosis after intestinal IRI, as well as damage to the liver and kidneys. Further elucidation of the mechanisms of protection may lead to advancements in the treatment of intestinal and multi-organ dysfunction following intestinal IRI.

## Methods

### Ethics Statements

This study was approved by the Animal Experimental Committee of Wenzhou Medical College. Animal care and all procedures were performed according to the guidelines for the care and use of laboratory animals.

### Animals

Male Sprague–Dawley rats (n = 50), weighing between 250**–**350 g, were used for this study. Rats were randomly divided into sham, control and study groups. In the sham group (n = 10), laparotomy was performed, but without aortic occlusion. In the control group (n = 10), after laparotomy the arteria mesenterica anterior was clamped for 45 min, followed by reperfusion for 24 h. In the study group, montelukast (0.2, 2 and 20 mg/kg) was administered intragastrically before the procedure (each group n = 10). The anaesthesia was maintained with intermittent delivery of hydral, and intraperitoneal cephalosporin (10 mg/kg) was administered before skin incision. The abdominal aorta was explored through a transperitoneal approach, retracting the intestines after surface cleaning of the surgical area and standard midline laparotomy. Intestinal ischemia was induced by clamping the arteria mesenterica anterior for 45 min. After surgery, the abdominal wall was sutured with 5/0 polypropylene suture thread. After 24 h, all animals were anaesthetised and sacrificed. Some distal ileum tissue and blood were extracted and stored at **−**80 °C until needed for further detection.

### Drug

Montelukast was purchased from the Simvastatin pharmacy company. The soluble form was prepared from oral tablets because we could not obtain the commercial soluble form. Ten tablets of montelukast (10 mg) were dissolved in 10 mL of ethanol. The solution was centrifuged for 5 min, and the supernatant was collected and filtered with a 0.2-mM filter. The resulting solution was concentrated by evaporation and reduced to a volume of 3 mL. The concentration of montelukast sodium was approximately 30 mg/mL.

### Histopathological Assessment

Paraffin sections (4-μm thickness) of intestinal tissue were prepared and stained with haematoxylin and eosin (H&E). Histopathological assessment was performed by a researcher blinded to the treatment groups. The slides were observed by light microscopy at 100× magnification, and the severity of mucosal injury was evaluated by using the scoring system published by Chiu *et al.*^26^. Grade 0 represented normal villi and grades 1–5 indicated increments of severity of injury (grade 1: mild development of subepithelial Gruenhagen spaces; grade 2 or 3: moderate (grade 2) or severe (grade 3) progressive lifting of the epithelial layer from the lamina propria; grade 4: completely denuded villi; grade 5: disintegration of the lamina propria). Kidney and liver tissue also underwent H&E examination.

### Real-time quantitative PCR

Real-time PCR was performed with SYBR Green PCR Master Mix (Applied Biosystems, Foster City, CA, USA) and an ABI Prism 7500 Sequence Detector (Applied Biosystems). Briefly, total RNA was isolated from intestinal tissue with Trizol reagent (Invitrogen, Life Technologies, Carlsbad, CA, USA), and complementary DNA was produced according to the manufacturer’s instructions for the transcriptor First Strand cDNA Synthesis Kit (Roche, Indianapolis, IN, USA). All samples were detected in triplicate and glyceraldehyde-3-phosphate dehydrogenase (GAPDH) was used as an internal control. The expression level of CysLTR1 mRNA was normalised to that of GAPDH and was expressed as a ratio relative to GAPDH.

### Western blot analysis

The protein expression of CysLTR1, Caspase-8 and Caspase-9 was detected by western blotting. Briefly, total protein was extracted, and the concentration of supernatants was measured using the BCA protein assay (Pierce). Aliquots of supernatants containing 100 μg of protein were electrophoresed on 5% (w/v) sodium dodecyl sulfate-polyacrylamide gels and transferred to polyvinylidene difluoride membrane. After blocking for 1 h with 5% (w/v) non-fat milk, the blots were incubated with primary antibody at 4 °C overnight. The membranes were washed in tris-buffered saline and incubated with the secondary antibody. After additional washing, the blots were exposed to films in a dark room. Protein bands were analysed with image analysis software (Quantity one, Bio-rad, Hercules, CA, USA). GAPDH served as the internal control, and the results were expressed as a ratio relative to GAPDH.

### Apoptosis detection using terminal dexynucleotidyl transferase(TdT)-mediated dUTP nick end labeling (TUNEL)

Apoptosis detection in tissue was performed using the Tetramethylrhodamine (TRITC) staining Apoptosis Detection Kit (Keygen Biotech, China) and fluorescence microscopy (BD Biosciences, San Jose, CA, USA). Briefly, cells were trypsinised, washed with Phosphate Buffered Saline (PBS), centrifuged, fixed for 20 min at room temperature, incubated with blocking solution containing 3% (v/v) H_2_O_2_ in methanol for 10 min at room temperature, and incubated in permeabilisation solution for 2 min on ice. Fifty microliters of TUNEL Reaction Mixture was added to samples and incubated for 60 min at 37 °C in the dark according to the manufacturer’s instructions. Last, samples were assessed using a fluorescence microscope at an excitation wavelength of 543 nm and an emission wavelength of 571 nm within 1 h.

### Plasma alanine aminotransferase (ALT) and aspartate aminotransferase (AST) levels after intestinal IRI

ALT and AST levels in rat plasma were measured at 3, 6, 12 and 24 h using the assay kit according to the manufacturer’s instructions (Thermo Fisher Scientific, Waltham, MA, USA).

### Neutrophil gelatinase associated lipocalin (NGAL) expression in the kidney

NGAL expression in the kidney was measured immunohistochemically. Paraffin sections were prepared, and tissue sections were deparaffinised, and hydrated in xylene and graded alcohol. The polyclonal anti-rabbit NGAL antibody (Invitrogen, Life Technologies, Carlsbad, CA, USA) was used for the immunohistochemical detection according to standard procedures. Briefly, paraffin sections were deparaffinized and heat treated with citrate buffer, and incubated with NGAL antibody for 1 h (dilution 1:100) and mixed with skimmed milk powder at 2% again to reduce unspecific staining. Then they were reacted with biotinylated secondary antibody (dilution 1:500) for 30 min. Negative controls were performed by omitting the primary antibody.

### Determination of IL-6 and TNF-α in plasma

After reperfusion, blood was collected into heparinised tubes and plasma was isolated via centrifugation (300 × *g* for 10 min). Plasma was snap frozen in liquid nitrogen and stored at **−**20 °C until assayed. Plasma IL-6 was measured by ELISA using the rat IL-6 ELISA kit (BD Biosciences), and Plasma TNF-α level was also measured by rat kit (Boster, WuHan, China).

### Data analysis

According to the relative study of intestinal IRI^18^with a standard of 0.05, and avoiding unnecessary waste, we used 10 rats in each group. Statistical comparisons were performed with the software package SPSS 16.0 (IBM Corp., Armonk, NY, USA). The distribution of data was analysed first, followed by a two-tailed Student’s t-test for comparisons between two groups, and one-way ANOVA plus Tukey’s *post hoc* multiple comparison test to compare multiple groups when data was normally distributed. The ordinal values of the Chiu scores were analysed by the Mann–Whitney nonparametric test. A *P* < 0.05 was considered statistically significant. All data are expressed as the mean ± SEM.

## Additional Information

**How to cite this article**: Wu, S. *et al.* The protective role of montelukast against intestinal ischemia-reperfusion injury in rats. *Sci. Rep.* **5**, 15787; doi: 10.1038/srep15787 (2015).
